# Mapping brain functional and structural abnormities in autism spectrum disorder: moving toward precision treatment

**DOI:** 10.1093/psyrad/kkac013

**Published:** 2022-11-09

**Authors:** Xujun Duan, Huafu Chen

**Affiliations:** School of Life Science and Technology, University of Electronic Science and Technology of China, Chengdu 610054, PR China; MOE Key Laboratory for Neuroinformation, High-Field Magnetic Resonance Brain Imaging Key Laboratory of Sichuan Province, University of Electronic Science and Technology of China, Chengdu 610054, PR China; School of Life Science and Technology, University of Electronic Science and Technology of China, Chengdu 610054, PR China; MOE Key Laboratory for Neuroinformation, High-Field Magnetic Resonance Brain Imaging Key Laboratory of Sichuan Province, University of Electronic Science and Technology of China, Chengdu 610054, PR China

**Keywords:** autism spectrum disorder, fMRI, brain function and structure, precision treatment

## Abstract

Autism spectrum disorder (ASD) is a formidable challenge for psychiatry and neuroscience because of its high prevalence, lifelong nature, complexity, and substantial heterogeneity. A major goal of neuroimaging studies of ASD is to understand the neurobiological underpinnings of this disorder from multi-dimensional and multi-level perspectives, by investigating how brain anatomy, function, and connectivity are altered in ASD, and how they vary across the population. However, ongoing debate exists within those studies, and neuroimaging findings in ASD are often contradictory. Over the past decade, we have dedicated to delineate a comprehensive and consistent mapping of the abnormal structure and function of the autistic brain, and this review synthesizes the findings across our studies reaching a consensus that the “social brain” are the most affected regions in the autistic brain at different levels and modalities. We suggest that the social brain network can serve as a plausible biomarker and potential target for effective intervention in individuals with ASD.

## Introduction

Autism spectrum disorder (ASD) is a complex neurodevelopmental conduction characterized by impairments in social communication and social interactions, along with the presence of repetitive patterns of behavior, restricted interests, and/or altered sensory responsivity to external stimuli (American Psychiatric Association, 2013). It has been almost 80 years since Leo Kanner first published his systematic description of early infantile autism in 1943, and over the ensuing years, the features Kanner described, lack of interest and lack of engagement in social contact, along with restricted and repetitive behaviors, continued to remain core features of the disorder (Harris, 2018). Up until 1980, “infantile autism” was added to the third edition of the Diagnostic and Statistical Manual of Mental Disorders (DSM-III), and later changed to “autistic disorder” in 1987 (Wolff, 2004). Under the latest released DSM-5, autism, Asperger's, and pervasive developmental disorder-not otherwise specified are grouped together as part of a broader diagnosis known as ASDs (American Psychiatric Association, 2013; Hull *et al*., 2016). In 2021, the US Centers for Disease Control and Prevention (CDC) reported that ∼1 in 44 children in USA is diagnosed with ASD, according to 2018 data, with markedly increasing prevalence showing rates among 8-year-old children from 1 in 110 for 2006 to 1 in 54 for 2016. The observed ASD prevalence rate was 0.29% in the population of 6- to 12-year-old children in China, based on a nationwide multi-center epidemiology study (Zhou *et al*., 2020), while a joint China-UK study has reported ∼1 in 100 school-aged children in China has ASD, which is in line with that found in the West (Sun *et al*., 2019). The potential factors that cause the increasing prevalence of ASD are suggested to be the environmental and genetic contributions, as well as the changes to diagnostic criteria, screening methods, and increased recognition of the disorder by parents and community (Rice *et al*., 2012).

Over the last several decades, the results of research and clinical work have helped us improving the understanding of this unique neurodevelopmental disorder with revisions in diagnostic classification, phenotype, and developmental models. Several theories have been proposed to elucidate and interpretate the symptomatology and underlying neural mechanism of ASD. The Amygdala Theory of autism suggested that the amygdala is one of the potential key neural regions in the pathophysiology of autism, and structural and functional abnormalities in the amygdala might result in the social behavior deficits in the developmental course of autism (Baron-Cohen *et al*., 2000); the social motivation theory of autism proposed that ASD can be construed as an extreme case of diminished social motivation which appear to be rooted in biological disruptions of the orbitofrontal-striatal-amygdala circuitry (Chevallier *et al*., 2012); the “Broken Mirror” theory of autism points out that the deficiency of action recognition, motion mimicry, theory of mind, empathy, and language in autistic people could be explained by the dysfunction of mirror neuron system (Hamilton, 2008).

Advances in neuroimaging technics have provided important insights into the neurobiological underpinning of this disorder. Positron emission topography was used to examine metabolic changes of the autistic brain (Zilbovicius *et al*., 2000; Zurcher *et al*., 2015; Murayama *et al*., 2022), and magnetic resonance imaging (MRI) was commonly employed to investigate morphological alterations of ASD (Brambilla *et al*., 2003; Del Casale *et al*., 2022). Functional MRI (fMRI), which measures the changes in blood-oxygen level-dependent contrast signals, was used to detect the abnormal brain activity of ASD during performance of a task or resting state (Choi *et al*., 2015; Nijhof *et al*., 2018). Furthermore, functional connectivity methods, which measure the synchronization of the activity between different brain regions, along with fiber tracking technics based on diffusion tensor imaging, provide further insights into the brain network dysfunction in individuals with ASD and verify the previously mentioned theories to a certain extent. However, ongoing debate exists within the neuroimaging literature over how structure and function are altered in the autistic brain, with several factors that might cause the inconsistent findings attributed to differences in demographic, heterogeneity in clinical manifestation, scanner parameters, in-scanner head motion, and methodology of data analysis.

Over the past decade, we have dedicated to delineate a comprehensive mapping of the abnormal structure and function of the autistic brain, and this review aimed to synthesize the findings across our research studies and address some key issues with regards to the neuroimaging biomarker of ASD. We summarize our findings into four fields: (i) brain dynamics in ASD, (ii) brain development in ASD, (iii) brain heterogeneity in ASD, and (iv) social brain of ASD, and finally discuss the potential future direction of using brain imaging-guided neural modulation in treatment of ASD.

## Brain Connectivity in ASD

Resting-state fMRI (rs-fMRI) has emerged as a powerful tool to assess the intrinsic organization of the human brain when no task is being performed. Over the last decades, rs-fMRI studies on ASD have reported disrupted brain connectivity in diverse brain systems in individuals with ASD. However, ongoing debate exists within the literature on how intrinsic connectivity is altered in the autistic brain, with reports of under-connectivity, over-connectivity, or a mixture of both, and the cause of these contradictory findings is still unclear. When we went over the literature, we found that, most of the previous studies assessing intrinsic functional connectivity based on the assumption that the resting-state connectivity patterns remain constant over time. However, theoretical models and empirical observations suggest that the functional connectivity patterns change dynamically in relation to the ongoing rhythmic activity of the human brain. This variation in brain connectivity can be revealed in different brain states by using dynamic functional connectivity techniques. Delineating the dynamic correlations across brain regions is of great importance to understand the basis of intrinsic functional organization and processing, and capturing nuanced time-varying properties of functional connections in the brain, thus reconciling inconsistencies in under- or over-connectivity in ASD.

Accumulating neuroimaging evidence suggests that abnormal functional connectivity of the default mode network (DMN) contributes to the social-communication deficits of ASD. Understanding the temporal dynamics may provide new insights into the dysfunction of the DMN and its association with social deficits in ASD. By using a sliding time window correlation, we found that young children with ASD exhibited decreased dynamic functional connectivity variance (less flexible) between the posterior cingulate cortex (PCC) and the right precentral gyrus, and the decreased variance was negatively correlated with social motivation and social relating of ASD (He *et al*., 2018). PCC is a critical node of the DMN, and it plays an important role in regulating attention between internally and externally focused thoughts and making responses to a rapidly changing environment, as well as planning for future actions (Leech *et al*., 2011; Pearson *et al*., 2011; Leech and Sharp, 2014). Specifically, the ability to plan future actions involves foreseeing future events and planning movements and movement sequences (Leech and Sharp, 2014). Previous studies reported that children with ASD split up chained motor acts into unrelated movements, thus resulting in a poor ability to plan for future actions (von Hofsten and Rosander, 2012). The ability to plan future actions influences the performance of each chained motor act, and previous studies have reported that the impaired development of social and communication abilities is related to abnormal motor function in children with ASD (Mostofsky *et al*., 2000). This was also verified in our study by the significant correlation between subscales related to motor function and social function. Therefore, abnormal dynamic functional connectivity between PCC and the precentral gyrus might suggest the disrupted interaction between high-level cognitive processing and motor function in young children with ASD.

Menon proposed a triple network model of psychopathology that posits that abnormal functional organization of the DMN, salience network and frontoparietal network (FPN) and their dynamic interactions underlie a wide range of psychopathologies, including autism (Menon, 2011). As a critical hub of the salience network, the anterior insula plays important roles in divers functions including consciousness, interoception, emotion, and interpersonal experience (Craig, 2002; Zaki *et al*., 2012). In particular, the right anterior insular (rAI) was proposed to act as a “causal outflow hub” in initiating the switching between internally (e.g. DMN) and externally (e.g. FPN) focused networks during cognitively demanding tasks and the resting state (Sridharan *et al*., 2008). By examining the dynamic connectivity of the right anterior insula, we found that significantly impaired connectivity was observed in ASD between the right anterior insula and DMN during the brain states relevant to socio-cognitive processing (Guo *et al*., 2018). From a dynamic perspective, these findings demonstrated aberrant interaction between the rAI and DMN across different states in the autistic brain, and provided new insights into the underlying neural mechanisms of social deficits in individuals with ASD.

Previous studies based on static functional connectivity analysis have demonstrated the abnormal intra- and interhemispheric pathways in ASD (Hahamy *et al*., 2015; Lee *et al*., 2016). By taking advantage of functional connectivity density (FCD) and sliding-window analysis, we demonstrated that both intra- and inter-hemispheric connections showed aberrant dynamic FCD (dFCD) variability in the social brain regions including the anterior cingulate cortex (ACC)/medial prefronal cortex (mPFC) and fusiform gyrus (FG)/interior temporal gyrus in autistic children compared with typically developing (TD) children, and the aberrant temporal variability of the contralateral dFCD predicted the severity of social communication impairments in autistic children (Guo *et al*., 2020). These findings indicate an abnormal temporal dynamic intra- and interhemispheric integration in brain regions within the social brain network of ASD, which might contribute to the impaired social processing in ASD.

The variance of dynamic functional connections was defined as the respective standard deviations of the dynamic functional connectivity strength across time, and it might reflect the flexibility/stability of brain networks. By investigating the dynamics of the whole-brain functional connectivity network, we demonstrated greater variance of widespread long-range dynamic functional connections in ASD, compared with TD, and the hyper-variant ASD connections were again associated with the social brain regions, including the mPFC, temporal pole, and ACC, suggesting the unstable transmission between those brain regions (Chen *et al*., 2017). The imbalanced excitation/inhibition theory of ASD suggested that the excess of excitation and loss of inhibition could result in enhanced “noise” of brain regions and imprecise brain activity in ASD (Nelson and Valakh, 2015), which might be associated with disturbed information transmission among the social brain regions and thus resulting social deficits of ASD.

## Brain Development in ASD

ASD undergoes an atypical trajectory of brain maturation that probably affects autistic symptoms across the lifespan (Ecker *et al*., 2015). Previous longitudinal and cross-sectional neuroimaging studies reported age-related abnormalities in ASD, suggesting an overgrowth in brain anatomy in early childhood but an accelerated decline during adolescence and young adulthood (Courchesne *et al*., 2011). Functional imaging studies also reported age-related abnormalities in functional connectivity of ASD, including atypical developmental trajectory of DMN (Wiggins *et al*., 2011), as well as the “social brain” network (Alaerts *et al*., 2015), and a developmental model was proposed of hyper-connectivity in children and hypo-connectivity in adolescents and adults (Uddin *et al*., 2013). All those findings suggested atypical structural and functional developmental trajectories across the lifespan in ASD, and highlighted the importance of investigating the neural etiology of ASD from a developmental perspective.

By using a large, multisite, resting-state fMRI dataset from the open-assess Autism Brain Imaging Data Exchange (ABIDE, http://fcon_1000.projects.nitrc.org/indi/abide/) repository, we have demonstrated the atypical developmental trajectory of local spontaneous brain activity in ASD, reported significant diagnosis-by-age interaction in the amplitude of low-frequency fluctuations (ALFF) of mPFC, and we also found that remarkable quadratic change of ALFF with increasing age in mPFC was presented in TC group but absent in ASD (Guo *et al*., 2017). Previous studies indicated that individuals with ASD had long-range under-connectivity and local over-connectivity (Courchesne and Pierce, 2005; Keown *et al*., 2013), but not all evidence supports this hypothesis. We posited that neurodevelopmental factors should be taken into consideration when assessing the distance-dependent connectivity abnormity in ASD. By using a three-way analysis of covariance on resting-state functional connectivity, we demonstrated a significant diagnosis-by-distance interaction in the DMN regions, and a diagnosis-by-age-by-distance interaction in orbitofrontal cortex, and suggesting a role of connection length in developmental changes of functional connectivity in ASD (Long *et al*., 2016).

**Figure 1:**
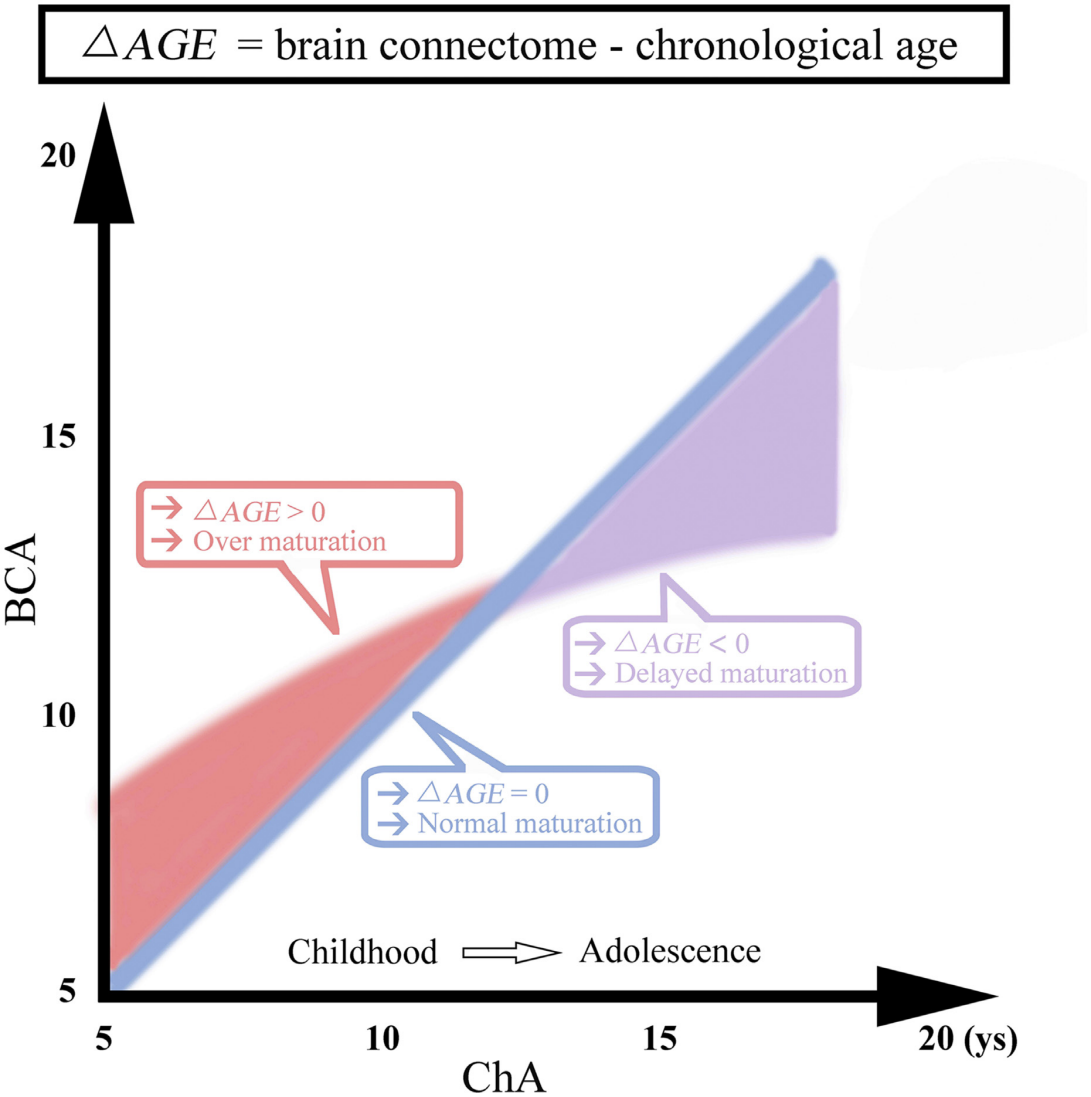
Schematic illustration of the relationship between BCA and ChA in ASD and TD. Blue line, BCA and ChA are highly correlated in TD. Red regime: BCA was higher than ChA in childhood ASD. Purple regime: BCA was lower than ChA in adolescent ASD. ys, years (He *et al*., 2020).

Age estimation obtained from brain connectomics might reflect the level of brain maturation along with neural development. By employing support vector regression, we estimated the brain connectome age (BCA) from structure–function connectomics constructed by white matter fiber tracking and resting-state functional connectivity. We found that BCA matched well with chronological age (ChA) in TD children and adolescents, but not in ASD. Specifically, our findings delineated an abnormal developmental trajectory of ASD with accelerated brain maturation in youth, followed by a delay of brain development starting at preadolescence (Fig. 1 ) (He *et al*., 2020).

The ABIDE team invested tremendous effort and dedicated to aggregating and sharing a mass of previously collected neuroimaging data sets from individuals with ASDs and matched TDs, greatly promoting the investigation of brain imaging of ASD and advancing knowledge of ASD neurobiology. However, ASD is an early-onset neurodevelopmental condition, and a potential limitation of using the ABIDE dataset for early-stage neurodevelopmental study is the age range of the ABIDE sample, which was between 5.8 and 64 years of age (Di Martino *et al*., 2014). Early childhood is widely recognized as an important period for brain development, due to the intense formation and fine-tuning of neural circuits (Courchesne *et al*., 2007). Depict the neuroanatomical development of ASD in early childhood may help understand the progression of the disorder in other stages of life, and facilitate early diagnosis and intervention. By recruiting young children with ASD ranging from 2 to 7 years of age, we aimed to map the abnormal grey matter development in the autistic brain during early childhood. By using volumetric-based methodology and causal structural covariance analysis with high-resolution structural MRI, we revealed a significant diagnosis-by-age interaction effect in the grey matter volume of the fusiform face area (FFA), and the grey matter development of the FFA in autism displayed altered influences on that of the social brain network regions. These findings indicated the atypical neurodevelopment of the FFA in the autistic brain during early childhood and highlighted abnormal developmental effects of this region on the social brain circuitry (Guo *et al*., 2021).

Although atypical subcortical neuroanatomy has been widely documented in individuals with ASD, the atypical coordinated neurodevelopment of these regions is barely known, especially in the autistic brain during early childhood. The perspective of coordinated neurodevelopment suggests that synchronous firing can induce synaptogenesis between neurons, thus building up use-dependent functional association between remote brain regions. Brain regions that grow together are highly correlated in morphometry, suggesting maturational coupling or structural covariance between them (Alexander-Bloch *et al*., 2013). By using structural covariance approach, we found that compared with TD children, children with ASD exhibited decreased structural covariation between the subcortical regions from different hemispheres, and increased structural covariation between adjacent regions. These results suggested decreased structural covariation between long-distance regions and excessive local covariation in subcortical structures in children with ASD, highlighting aberrant developmental coordination or synchronized maturation between subcortical regions that play crucial roles in social cognition and behavior in ASD (Duan *et al*., 2020). Synchronous maturation intensity was also evaluated by individual-based morphological brain networks built from structural MRI data, which characterizes inter-regional similarities in several morphological aspects of gray matter. We found that the morphological brain network in the autistic developmental brain is inefficient in segregating and distributing information (lower values of small-worldness in ASD children, compared with matched TD children). The results also highlight the crucial role of abnormal morphological connectivity patterns of the cortico-striatum-thalamic-cortical circuitry in the socio-cognitive deficits of ASD (He *et al*., 2021).

## Decomposing Brain Heterogeneity in ASD

Individuals with ASD exhibit multilevel heterogeneity across genetics (Baker and Jeste, 2015; Geschwind and State, 2015), brain systems (Tang *et al*., 2020), and symptomatology (Huerta and Lord, 2012). This heterogeneity has become the largest obstacle in the understanding and treatment of ASD. The identification of homogeneous subgroups of ASD may help depict this heterogeneity, thus facilitating diagnosis and individualized treatment. Structural MRI is a noninvasive tool with high spatial resolution for probing brain structural organization. Previous studies based on structural MRI have identified two to four ASD subtypes (Hong *et al*., 2020), despite varying in methodology. However, subtype clustering features used in previous studies were either based on previous knowledge (selected ROIs) or high-dimensional whole-brain data, lacking quantitative and individualized metrics for delineating the heterogeneity of brain structure in ASD.

By using structural MRI data from the large-sample ABIDE dataset, we revealed evidence for three ASD subtypes with distinct neuroanatomical difference patters (Chen *et al*., 2019). In this study, individual neuroanatomical difference patterns for each ASD individual were calculated by assessing the difference between each participant with ASD and 20 TD from the same site with the most similar age and FIQ to the corresponding ASD participant. A data-driven clustering method was next used to stratify individuals with ASD into three subtypes with different grey matter patterns, and among these subtypes, different clinical symptom severity and brain functional connectivity were revealed. After dividing the ASD group into different subtypes, accuracy of classification between two out of the three ASD subtypes and HC was improved from 58% based on the entire ASD dataset to 79% on the subtypes, suggesting the potential benefit of subdividing ASD for the development of brain-based biomarkers of the disorder.

**Figure 2:**
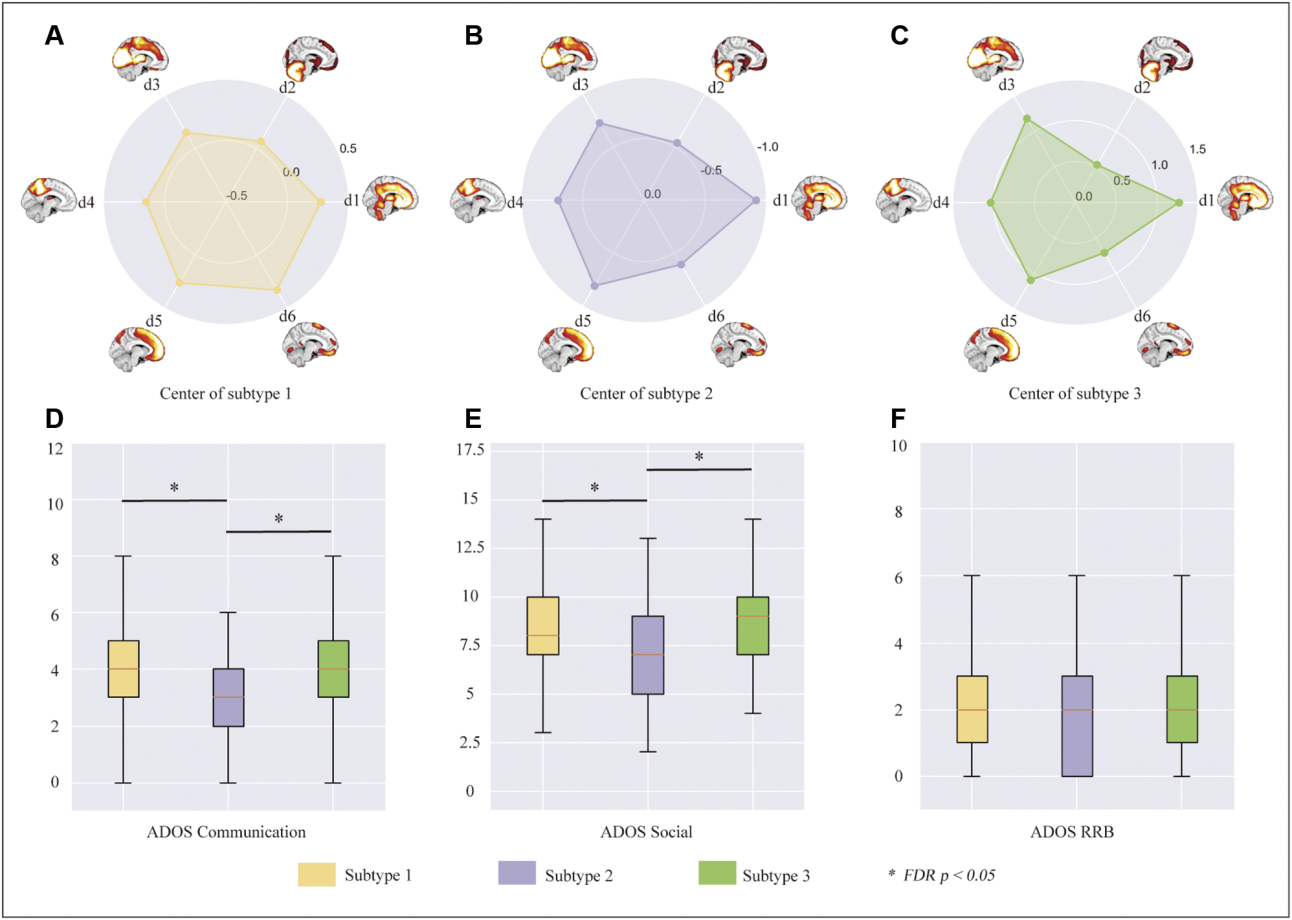
Three ASD subtypes with distinct neuroanatomical deviation patterns were identified. (A–C) Subtypes 1 and 3 showed positive deviations of the six grey matter factors, whereas ASD Subtype 2 showed negative deviations. (D–F) Distinct clinical manifestations in social communication deficits were identified among the three subtypes. *Significant between-group differences after false discovery rate (FDR) correction (*P* < 0.05, two-sample *t*-test) (Shan *et al*., 2022).

The previously mentioned study showed a way to compute an individual structural difference map by matching a group of most similar healthy control participants for each individual with ASD; however, the generalizability and clinical use of the approach is limited due to the matching strategy. To develop a parameterized, low-dimensional, and individualized metric to delineate heterogeneity of brain structure in ASD, we used the nonnegative matrix factorization to decompose the whole-brain gray matter into distinct factors and their corresponding weights, and then used normative modeling to estimate the factor deviations. The normative model is similar to the height and weight development curves used in pediatrics, which provide statistical inferences to delineate individual difference on quantitative biological measures, based on the extent to which each individual deviates from the reference population (i.e. the normative range) (Cole, 2012; Wang, 2022). Conventional case-control studies examine patients versus control participants at a group mean level that assume an “average patient,” while normative models provide individualized and parameterized metrics for each patient compared to normative ranges, parsing and capitalizing individual differences on individual level. In this study, six latent grey matter factors and their corresponding weights were obtained using nonnegative matrix factorization, individual deviation of the six latent factors were obtained using normative modeling, and finally three subtypes with distinct neuroanatomical deviation patterns were identified by clustering analysis, among which distinct clinical manifestations in social communication deficits were identified (Fig. 2) (Shan *et al*., 2022). This study presents a framework for understanding inter-individual differences in neurobiology, and takes an important step forward in parsing the heterogeneity of ASD, potentially leading to future biomarker development.

## Social Brain of ASD: A Potential Targeting Circuit for Precision Treatment

Over the past several decades, several theories have been put forward to account for the pervasive social impairments of ASD. Among the various attempts, the social cognition theories have become the most prominent ones. Social cognition and social behavior are subserved by a network of brain regions including the amygdala, the orbitofrontal cortex, the superior temporal sulcus and gyrus, mPFC, ACC, the temporo-parietal junction, the anterior insula, the hippocampus, the anterior temporal lobes, and the fusiform gyrus. Based on their involvement in facial recognition, mental state attribution, emotional awareness, self-reflection, empathy, and social interaction and social judgment, those brain regions were called “social brain” (Blakemore, 2008; Gotts *et al*., 2012; Kennedy and Adolphs, 2012). When we refer back to our section (ii), Brain dynamics in ASD, it is not surprising to find out that most of the findings related to abnormal brain dynamics were associated with the social brain regions.

Among the social brain regions, the amygdala is the one that gets the most attention. The amygdala has been identified as a central component in the neural circuits underlying social behavior and social cognition, especially in social-cued emotional processing (Phelps and LeDoux, 2005), and it plays an important role in directing internal attention to biologically relevant stimuli, such as social information delivered by eyes, faces, or biological motion (Chevallier *et al*., 2012), and attaches emotional value to faces, enabling us to recognize expressions such as fear and trustworthiness (Frith, 2007). The amygdala theory of autism was proposed that the amygdala is one of potential key neural regions in the pathophysiology of autism, and structure and function abnormalities in the amygdala might result in the social deficits in the developmental course of ASD (Baron-Cohen *et al*., 2000; Dziobek *et al*., 2006). By using two independent datasets from the ABIDE dataset, we conducted seed-based resting-state functional connectivity analyses to explore the functional connectivity patterns associated with the amygdala in adolescents with ASD. We found that compared with typically developing controls, adolescents with ASD showed consistent decreased functional connectivity between the amygdala and subcortical regions in both datasets, including the bilateral thalamus and right putamen. These findings support the Amygdala Theory of Autism by demonstrating impaired functional connectivity of the amygdala and its association with social deficits in ASD (Guo *et al*., 2016).

In addition to the amygdala, more and more neuroimaging studies have reported that social behavior deficits in children with ASD are commonly attributed to the dysfunction of the widely distributed social brain regions. Understanding the dynamic interaction within the social brain network and its association with social deficits is of great importance in understanding the abnormal social behavior of ASD. To evaluate the dynamic interaction among the social brain regions, we employed multivariate Granger causality analysis to explore the intrinsic effective connectivity within the “social brain” of ASD, and we found significantly attenuated effective connectivity from the mPFC to the bilateral amygdala in children with the ASD compared with TDC. The mPFC is a key node of the mentalizing network, and it is substantially involved in complex social tasks, such as inferring others’ intentions (theory of mind) (Spunt *et al*., 2011), and it provides contextual and experiential input into the amygdala to regulate emotional responses triggered by the amygdala, which is subsequently used to interpret social stimuli and prepare behavioral and emotional responses (Adolphs, 2003). The attenuated mPFC-amygdala pathway in the social brain might related to impaired modulation of the prefrontal cortex to the amygdala in regulating social emotional responses, thus resulting in core social deficits in individuals with ASD.

Based on the consistent findings of the social brain network dysfunction in ASD from our studies, we suggest that the social brain network can serve as a biomarker and target for intervention in individuals with ASD. In the past decade, the emerging noninvasive brain stimulation techniques have been verified as new therapeutic options to modify the pathological neuroplasticity and provide the potential to complement the behavioral therapy for psychiatric disorders including ASD (Oberman *et al*., 2016). Several brain regions have been commonly targeted to improve different symptom domains, including dorsal lateral prefrontal cortex (DLPFC) to improve irritability, repetitive behaviors, and executive functioning, primary and supplementary motor regions to improve sensory and motor behavior, medial prefrontal cortex to improve mentalizing. However, the ideal stimulation target was often ambiguous in those interventions, and demonstrated only moderate effects in reducing motor, sensory and executive symptoms, and very limited improvement on the core symptoms of social and communication in ASD. Based on our previous findings, we suggest that key regions in the social brain circuitry including the amygdala, the precuneus, and the frontal-insular cortex can be targeted in neural stimulation therapy to improve the social and communication deficits in ASD. Personalized targets can be identified by neuro-navigation techniques, and deep brain regions can be targeted by stimulating the sites on the cortex that demonstrate strong functional connectivity with the deep regions, based on the notion that magnetic stimulation can be delivered through resting-state networks (Fox *et al*., 2014). However, future studies on clinical practice are desirable to verify the inference by targeting the social brain network and symptom domains of ASD.

## Conclusion and Future Directions

We delineated a comprehensive mapping of the abnormal structure and function of the autistic brain, from perspectives of brain dynamics, brain development, and brain heterogeneity, and addressed the key issues with regards to the neuroimaging biomarker of ASD. We found that the “social brain” regions are the most affected in the autistic brain at different levels and modalities, supporting the cognitive theories of ASD from the neuroimaging aspect. The interplay between cognitive theories of ASD and findings from neuroimaging studies has greatly improved our understanding of the neural pathology of ASD, and one of the future directions for neuroimaging studies should be put forward for clinical use, either developing a robust classifier based on a reliable neuroimaging biomarker to assist the diagnosis, or determine potential targets for precision treatment of ASD. Although some progress has been made, there is still a long way to go.

## References

[bib1] Adolphs R (2003) Cognitive neuroscience of human social behaviour. Nat Rev Neurosci. 4:165–78. 12612630 10.1038/nrn1056

[bib2] Alaerts K, Nayar K, Kelly C et al. (2015) Age-related changes in intrinsic function of the superior temporal sulcus in autism spectrum disorders. Soc Cogn Affect Neurosci. 10:1413–23. 25809403 10.1093/scan/nsv029 PMC4590540

[bib3] Alexander-Bloch A, Giedd JN, Bullmore E (2013) Imaging structural co-variance between human brain regions. Nat Rev Neurosci. 14:322–36. 23531697 10.1038/nrn3465 PMC4043276

[bib4] American Psychiatric Association. (2013) The Diagnostic and Statistical Manual of Mental Disorders: DSM 5. Arlington (VA): American Psychiatric Publication.

[bib5] Baker E, Jeste SS (2015) Diagnosis and management of autism spectrum disorder in the era of genomics: rare disorders can pave the way for targeted treatments. Pediatr Clin North Am. 62:607–18. 26022165 10.1016/j.pcl.2015.03.003 PMC4449456

[bib6] Baron-Cohen S, Ring HA, Bullmore ET et al. (2000) The amygdala theory of autism. Neurosci Biobehav Rev. 24:355–64. 10781695 10.1016/s0149-7634(00)00011-7

[bib7] Blakemore SJ (2008) The social brain in adolescence. Nat Rev Neurosci. 9:267–77. 18354399 10.1038/nrn2353

[bib8] Brambilla P, Hardan A, di Nemi SU et al. (2003) Brain anatomy and development in autism: review of structural MRI studies. Brain Res Bull. 61(6):557–69. 14519452 10.1016/j.brainresbull.2003.06.001

[bib9] Chen H, Nomi JS, Uddin LQ et al. (2017) Intrinsic functional connectivity variance and state-specific under-connectivity in autism. Hum Brain Mapp. 38:5740–55. 28792117 10.1002/hbm.23764 PMC5783325

[bib10] Chen H, Uddin LQ, Guo X et al. (2019) Parsing brain structural heterogeneity in males with autism spectrum disorder reveals distinct clinical subtypes. Hum Brain Mapp. 40:628–37. 30251763 10.1002/hbm.24400 PMC6865602

[bib11] Chevallier C, Kohls G, Troiani V et al. (2012) The social motivation theory of autism. Trends Cogn Sci. 16:231–9. 22425667 10.1016/j.tics.2012.02.007 PMC3329932

[bib12] Choi US, Kim SY, Sim HJ et al. (2015) Abnormal brain activity in social reward learning in children with autism spectrum disorder: an fMRI study. Yonsei Med J. 56:705–11. 25837176 10.3349/ymj.2015.56.3.705 PMC4397440

[bib13] Cole TJ (2012) The development of growth references and growth charts. Ann Hum Biol. 39:382–94. 22780429 10.3109/03014460.2012.694475 PMC3920659

[bib14] Courchesne E, Campbell K, Solso S (2011) Brain growth across the life span in autism: age-specific changes in anatomical pathology. Brain Res. 1380:138–45. 20920490 10.1016/j.brainres.2010.09.101 PMC4500507

[bib15] Courchesne E, Pierce K (2005) Why the frontal cortex in autism might be talking only to itself: local over-connectivity but long-distance disconnection. Curr Opin Neurobiol. 15:225–30. 15831407 10.1016/j.conb.2005.03.001

[bib16] Courchesne E, Pierce K, Schumann CM et al. (2007) Mapping early brain development in autism. Neuron. 56:399–413. 17964254 10.1016/j.neuron.2007.10.016

[bib17] Craig AD (2002) How do you feel? Interoception: the sense of the physiological condition of the body. Nat Rev Neurosci. 3:655–66. 12154366 10.1038/nrn894

[bib18] Del Casale A, Ferracuti S, Alcibiade A et al. (2022) Neuroanatomical correlates of autism spectrum disorders: a meta-analysis of structural magnetic resonance imaging (MRI) studies. Psychiatry Res Neuroimaging. 325:111516. 35882091 10.1016/j.pscychresns.2022.111516

[bib19] Di Martino A, Yan CG, Li Q et al. (2014) The autism brain imaging data exchange: towards a large-scale evaluation of the intrinsic brain architecture in autism. Mol Psychiatry. 19:659–67. 23774715 10.1038/mp.2013.78 PMC4162310

[bib20] Duan X, Wang R, Xiao J et al. (2020) Subcortical structural covariance in young children with autism spectrum disorder. Prog Neuropsychopharmacol Biol Psychiatry. 99:109874. 31981719 10.1016/j.pnpbp.2020.109874

[bib21] Dziobek I, Fleck S, Rogers K et al. (2006) The ‘amygdala theory of autism’ revisited: linking structure to behavior. Neuropsychologia. 44:1891–9. 16566949 10.1016/j.neuropsychologia.2006.02.005

[bib22] Ecker C, Bookheimer SY, Murphy DG (2015) Neuroimaging in autism spectrum disorder: brain structure and function across the lifespan. Lancet Neurol. 14:1121–34. 25891007 10.1016/S1474-4422(15)00050-2

[bib23] Fox MD, Buckner RL, Liu H et al. (2014) Resting-state networks link invasive and noninvasive brain stimulation across diverse psychiatric and neurological diseases. Proc Natl Acad Sci USA. 111:E4367–75. 25267639 10.1073/pnas.1405003111 PMC4205651

[bib24] Frith CD (2007) The social brain?. Philos Trans R Soc Lond B Biol Sci. 362:671–8. 17255010 10.1098/rstb.2006.2003 PMC1919402

[bib25] Geschwind DH, State MW (2015) Gene hunting in autism spectrum disorder: on the path to precision medicine. Lancet Neurol. 14:1109–20. 25891009 10.1016/S1474-4422(15)00044-7 PMC4694565

[bib26] Gotts SJ, Simmons WK, Milbury LA et al. (2012) Fractionation of social brain circuits in autism spectrum disorders. Brain. 135:2711–25. 22791801 10.1093/brain/aws160 PMC3437021

[bib27] Guo X, Chen H, Long Z et al. (2017) Atypical developmental trajectory of local spontaneous brain activity in autism spectrum disorder. Sci Rep. 7:39822. 28057930 10.1038/srep39822 PMC5216408

[bib28] Guo X, Duan X, Chen H et al. (2020) Altered inter- and intrahemispheric functional connectivity dynamics in autistic children. Hum Brain Mapp. 41:419–28. 31600014 10.1002/hbm.24812 PMC7268059

[bib29] Guo X, Duan X, Long Z et al. (2016) Decreased amygdala functional connectivity in adolescents with autism: a resting-state fMRI study. Psychiatry Res Neuroimaging. 257:47–56. 27969061 10.1016/j.pscychresns.2016.10.005

[bib30] Guo X, Duan X, Suckling J et al. (2018) Partially impaired functional connectivity states between right anterior insula and default mode network in autism spectrum disorder. Hum Brain Mapp. 10.1002/hbm.24447 PMC6865537 30367744

[bib31] Guo X, Duan X, Suckling J et al. (2021) Mapping progressive gray matter alterations in early childhood autistic brain. Cereb Cortex. 31:1500–10. 33123725 10.1093/cercor/bhaa304 PMC7869087

[bib32] Hahamy A, Behrmann M, Malach R (2015) The idiosyncratic brain: distortion of spontaneous connectivity patterns in autism spectrum disorder. Nat Neurosci. 18:302–9. 25599222 10.1038/nn.3919

[bib33] Hamilton AF (2008) Emulation and mimicry for social interaction: a theoretical approach to imitation in autism. Q J Exp Psychol. 61:101–15. 10.1080/17470210701508798 18038342

[bib34] Harris J (2018) Leo Kanner and autism: a 75-year perspective. Int Rev Psychiatry. 30:3–17. 29667863 10.1080/09540261.2018.1455646

[bib35] He C, Chen H, Uddin LQ et al. (2020) Structure-function connectomics reveals aberrant developmental trajectory occurring at preadolescence in the autistic brain. Cereb Cortex. 30:5028–37. 32377684 10.1093/cercor/bhaa098 PMC7391416

[bib36] He C, Chen Y, Jian T et al. (2018) Dynamic functional connectivity analysis reveals decreased variability of the default-mode network in developing autistic brain. Autism Res. 11:1479–93. 30270547 10.1002/aur.2020

[bib37] He C, Cortes JM, Kang X et al. (2021) Individual-based morphological brain network organization and its association with autistic symptoms in young children with autism spectrum disorder. Hum Brain Mapp. 42:3282–94. 33934442 10.1002/hbm.25434 PMC8193534

[bib38] Hong SJ, Vogelstein JT, Gozzi A et al. (2020) Toward neurosubtypes in autism. Biol Psychiatry. 88:111–28. 32553193 10.1016/j.biopsych.2020.03.022

[bib39] Huerta M, Lord C (2012) Diagnostic evaluation of autism spectrum disorders. Pediatr Clin North Am. 59:103–11., xi. 22284796 10.1016/j.pcl.2011.10.018 PMC3269006

[bib40] Hull JV, Dokovna LB, Jacokes ZJ et al. (2016) Resting-state functional connectivity in autism spectrum disorders: a review. Front Psychiatry. 7:205. 28101064 10.3389/fpsyt.2016.00205 PMC5209637

[bib41] Kennedy DP, Adolphs R (2012) The social brain in psychiatric and neurological disorders. Trends Cogn Sci. 16:559–72. 23047070 10.1016/j.tics.2012.09.006 PMC3606817

[bib42] Keown CL, Shih P, Nair A et al. (2013) Local functional overconnectivity in posterior brain regions is associated with symptom severity in autism spectrum disorders. Cell Rep. 5:567–72. 24210815 10.1016/j.celrep.2013.10.003 PMC5708538

[bib43] Lee JM, Kyeong S, Kim E et al. (2016) Abnormalities of inter- and intra-hemispheric functional connectivity in autism spectrum disorders: a study using the autism brain imaging data exchange database. Front Neurosci. 10:191. 27199653 10.3389/fnins.2016.00191 PMC4853413

[bib44] Leech R, Kamourieh S, Beckmann CF et al. (2011) Fractionating the default mode network: distinct contributions of the ventral and dorsal posterior cingulate cortex to cognitive control. J Neurosci. 31:3217–24. 21368033 10.1523/JNEUROSCI.5626-10.2011 PMC6623935

[bib45] Leech R, Sharp DJ (2014) The role of the posterior cingulate cortex in cognition and disease. Brain. 137:12–32. 23869106 10.1093/brain/awt162 PMC3891440

[bib46] Long Z, Duan X, Mantini D et al. (2016) Alteration of functional connectivity in autism spectrum disorder: effect of age and anatomical distance. Sci Rep. 6:26527. 27194227 10.1038/srep26527 PMC4872225

[bib47] Menon V (2011) Large-scale brain networks and psychopathology: a unifying triple network model. Trends Cogn Sci. 15:483–506. 21908230 10.1016/j.tics.2011.08.003

[bib48] Mostofsky SH, Goldberg MC, Landa RJ et al. (2000) Evidence for a deficit in procedural learning in children and adolescents with autism: implications for cerebellar contribution. J Int Neuropsychol Soc. 6:752–9. 11105465 10.1017/s1355617700677020

[bib49] Murayama C, Iwabuchi T, Kato Y et al. (2022) Extrastriatal dopamine D2/3 receptor binding, functional connectivity, and autism socio-communicational deficits: a PET and fMRI study. Mol Psychiatry. 27:2106–13. 35181754 10.1038/s41380-022-01464-3

[bib50] Nelson SB, Valakh V (2015) Excitatory/inhibitory balance and circuit homeostasis in autism spectrum disorders. Neuron. 87:684–98. 26291155 10.1016/j.neuron.2015.07.033 PMC4567857

[bib51] Nijhof AD, Bardi L, Brass M et al. (2018) Brain activity for spontaneous and explicit mentalizing in adults with autism spectrum disorder: an fMRI study. Neuroimage Clin. 18:475–84. 29876255 10.1016/j.nicl.2018.02.016 PMC5987841

[bib52] Oberman LM, Enticott PG, Casanova MF et al. (2016) Transcranial magnetic stimulation in autism spectrum disorder: challenges, promise, and roadmap for future research. Autism Res. 9:184–203. 26536383 10.1002/aur.1567 PMC4956084

[bib53] Pearson JM, Heilbronner SR, Barack DL et al. (2011) Posterior cingulate cortex: adapting behavior to a changing world. Trends Cogn Sci. 15:143–51. 21420893 10.1016/j.tics.2011.02.002 PMC3070780

[bib54] Phelps EA, LeDoux JE (2005) Contributions of the amygdala to emotion processing: from animal models to human behavior. Neuron. 48:175–87. 16242399 10.1016/j.neuron.2005.09.025

[bib55] Rice CE, Rosanoff M, Dawson G et al. (2012) Evaluating changes in the prevalence of the autism spectrum disorders (ASDs). Public Health Rev. 34:1–22. 26236074 10.1007/BF03391685 PMC4520794

[bib56] Shan X, Uddin LQ, Xiao J et al. (2022) Mapping the heterogeneous brain structural phenotype of autism spectrum disorder using the normative model. Biol Psychiatry. 91:967–76. 35367047 10.1016/j.biopsych.2022.01.011

[bib57] Spunt RP, Satpute AB, Lieberman MD (2011) Identifying the what, why, and how of an observed action: an fMRI study of mentalizing and mechanizing during action observation. J Cogn Neurosci. 23:63–74. 20146607 10.1162/jocn.2010.21446

[bib58] Sridharan D, Levitin DJ, Menon V (2008) A critical role for the right fronto-insular cortex in switching between central-executive and default-mode networks. Proc Natl Acad Sci. 105:12569–74. 18723676 10.1073/pnas.0800005105 PMC2527952

[bib59] Sun X, Allison C, Wei L et al. (2019) Autism prevalence in China is comparable to Western prevalence. Molecular Autism. 10:7. 30858963 10.1186/s13229-018-0246-0 PMC6394100

[bib60] Tang S, Sun N, Floris DL et al. (2020) Reconciling dimensional and categorical models of autism heterogeneity: a brain connectomics and behavioral study. Biol Psychiatry. 87:1071–82. 31955916 10.1016/j.biopsych.2019.11.009

[bib61] Uddin LQ, Supekar K, Menon V (2013) Reconceptualizing functional brain connectivity in autism from a developmental perspective. Front Hum Neurosci. 7:458. 23966925 10.3389/fnhum.2013.00458 PMC3735986

[bib62] von Hofsten C, Rosander K (2012) Perception-action in children with ASD. Front Integr Neurosci. 6:115. 23248590 10.3389/fnint.2012.00115 PMC3520024

[bib63] Wang F (2022) Disentangling the heterogeneity of autism spectrum disorder using normative modeling. Biol Psychiatry. 91:920–1. 35589313 10.1016/j.biopsych.2022.03.005

[bib64] Wiggins JL, Peltier SJ, Ashinoff S et al. (2011) Using a self-organizing map algorithm to detect age-related changes in functional connectivity during rest in autism spectrum disorders. Brain Res. 1380:187–97. 21047495 10.1016/j.brainres.2010.10.102 PMC3050117

[bib65] Wolff S (2004) The history of autism. Eur Child Adolesc Psychiatry. 13:201–8. 15365889 10.1007/s00787-004-0363-5

[bib66] Zaki J, Davis JI, Ochsner KN (2012) Overlapping activity in anterior insula during interoception and emotional experience. Neuroimage. 62:493–9. 22587900 10.1016/j.neuroimage.2012.05.012 PMC6558972

[bib67] Zhou H, Xu X, Yan W et al. (2020) Prevalence of autism spectrum disorder in China: a nationwide multi-center population-based study among children aged 6 to 12 years. Neurosci Bull. 36:961–71. 32607739 10.1007/s12264-020-00530-6 PMC7475160

[bib68] Zilbovicius M, Boddaert N, Belin P et al. (2000) Temporal lobe dysfunction in childhood autism: a PET study. Positron emission tomography. Am J Psychiatry. 157:1988–93. 11097965 10.1176/appi.ajp.157.12.1988

[bib69] Zurcher NR, Bhanot A, McDougle CJ et al. (2015) A systematic review of molecular imaging (PET and SPECT) in autism spectrum disorder: current state and future research opportunities. Neurosci Biobehav Rev. 52:56–73. 25684726 10.1016/j.neubiorev.2015.02.002

